# USA and Portuguese Young Adolescents’ Perceived Qualities and Satisfaction in Their Relationships with Mothers, Fathers and Best-Friends

**DOI:** 10.3390/children9010026

**Published:** 2021-12-31

**Authors:** Maryse Guedes, Olívia Ribeiro, Miguel Freitas, Kenneth H. Rubin, António J. Santos

**Affiliations:** 1 William James Center for Research, ISPA-Instituto Universitário, 1100-304 Lisboa, Portugal; oribeiro@ispa.pt (O.R.); MFreitas@ispa.pt (M.F.); asantos@ispa.pt (A.J.S.); 2 Human Development and Quantitative Methodology, University of Maryland, College Park, MD 20742, USA; krubin@umd.edu

**Keywords:** young adolescents, close relationships, relationship positive and negative qualities, satisfaction

## Abstract

Background: Few researchers have examined young adolescents’ perceived qualities and satisfaction in their relationships with their mothers, fathers and best friends simultaneously, using a cross-cultural perspective. This study aimed to compare the perceived qualities and satisfaction of USA and Portuguese adolescents in their relationships with their parents and best friends and to examine the influence of perceived relationship qualities on the satisfaction of young adolescents with their close relationships. Methods: The sample consisted of 347 USA adolescents (170 boys, 177 girls) and 360 Portuguese adolescents (176 boys, 184 girls) who completed the *Network of Relationships Inventory Social Provision Version* to assess perceived support, negativity, power balance and satisfaction in their relationships with their mothers, fathers and same-sex best friends. Results: Adolescents from both countries perceived their relationships with parents to be more negative and imbalanced in power than their relationships with friends, but the magnitude of differences was greater in the USA. Furthermore, USA adolescents reported higher satisfaction in their relationships with friends than in their relationships with parents. Country differences in the concomitants of relationship satisfaction were found. Conclusions: These findings support the notion that young adolescents’ perceived qualities and satisfaction in close relationships may differ depending on cultural norms.

## 1. Introduction

As children make the transition to early adolescence, their close interpersonal relationships become increasingly complex, diverse and extensive [1,2]. Although parent-child relationships remain important [3,4], with increasing age, young adolescents begin to devote an increased amount of time interacting with peers and developing close, dyadic friendships [5] and to attribute to these extra-familial relationships many of the same functions that were exclusive to their relationships with parents during childhood [6]. In part, the transition into adolescence brings with it changes in sociocultural expectations for relating to others [1]. Given the overarching roles played by parents and same-sex friends in adolescent development [7], understanding how youth from different cultures perceive the functional features of their relationships with mothers, fathers and best friends is essential to develop evidence-based interventions that can promote healthy socioemotional adjustment. 

### 1.1. Perceived Qualities and Satisfaction in Close Relationships during Early Adolescence

The functional features of close relationships encompass both positive and negative qualities [8,9]. With respect to positive qualities, the social needs perspective [10] establishes that individuals typically seek a set of socially supportive features in their close relationships, such as: (1) attachment, that is, affection, security and a sense that one can disclose intimate information with a relationship partner; (2) reliable alliance, or a belief that the relationship partner can be depended upon; (3) enhancement of worth, that is, knowing that the relationship partner affirms one’s competence, value and sense of self; (4) social integration involving companionship and the mutual sharing of experience; (5) guidance, that is, believing that the relationship partner can be counted on to provide tangible aid and advice; and (6) opportunity for nurturance, or, taking care of others [8,11,12]. Beyond the aforementioned positive qualities that are organized along a dimension of social support, close relationships also vary along a dimension of power distribution [12], which reflects the extent to which each relationship is viewed as vertical/horizontal or less symmetrical and egalitarian in power balance [13]. In addition to power balance, close relationships can also be characterized by relationship-straining features, namely, conflict and antagonism [12].

Nevertheless, few researchers have examined the perceptions of young adolescents about the functional features of their relationships with their mothers, fathers and best friends simultaneously, using similar sets of items [14], and most of them have primarily focused on the perspectives of youth who live in North American cultures [15] during the decade of 1990. The few studies that have examined the functional features of close relationships during early adolescence, using similar sets of items, have found that young adolescents typically view their parents and best friends as the most frequent providers of support but report that they respond to different social needs [12,16,17,18]. Whereas parents are seen to provide more affection, reliable alliance, enhancement of worth and instrumental support [12], best friends are viewed as providing greater companionship, nurturance and intimacy than their parents [18]. With respect to power balance and negative interactions, relationships with parents have been rated as higher on the dimensions of power and negativity than relationships with friends [12,17,19].

Differences between the qualities of mother-child and father-child relationships during early adolescence have been less consensual. Whereas some researchers have reported non-significant differences between mother-child and father-child relationships in terms of positive qualities (e.g., affection and reliance, [18] power [19]), others have found that ratings of support from the same-sex parent are higher than those from the opposite-sex parent [12].

Researchers have also found that perceived relationship qualities vary depending on young adolescents’ sex. Girls typically report more positive qualities in their relationships with their mothers and best friends than boys, whereas boys typically perceive more positive qualities in their relationships with their fathers than girls [2,12,20,21]. The findings concerning negative interactions and power have been mixed. Whereas some studies report a lack of sex differences [19], others have found that girls perceive less of a power dynamic in their relationships than boys [17]. Furthermore, in some studies, boys, relative to girls, perceive their relationships with their fathers to fall along a more vertical, hierarchical plane of dominance and their relationships with their best friends to be more conflicted [2,20].

The way in which young adolescents evaluate the above-noted positive and negative qualities in their close relationships may influence the degree to which they are satisfied with those relationships [22]. Prior research conducted with North American samples has found that young adolescents typically report being more satisfied with their relationships with mothers, followed by fathers and then their best friends [12,18]. With respect to sex differences, [18] found that boys were equally satisfied with their relationships with mothers, fathers and friends, whereas girls were equally satisfied with their relationships with same-sex best friends and mothers but less satisfied with their relationships with fathers. Furthermore, higher levels of supportive relationship provisions and lower levels of negative qualities were found to be associated with higher levels of satisfaction in USA adolescents’ relationships [7].

Despite the contributions of the aforementioned research, it remains relatively unknown whether the perceptions of young adolescents about the relationship qualities and their associations with satisfaction in close relationships vary depending on the norms and value systems of specific cultures [7].

### 1.2. Culture and Perceived Qualities of Close Relationships

Culture can be defined as “the set of distinctive patterns of beliefs and behaviors that are shared by a group of people and that serve to regulate their daily living” ([23], p. 212). Cultural beliefs and norms help interpret the types and ranges of relationships that are likely or permissible [7]. As such, culture is a primary ecological context that shapes parental socialization goals, beliefs and practices of child-rearing [22,24,25,26] and influences the social norms and norm-related interpersonal perceptions, evaluations and reactions that regulate family and peer relationships [7,27].

In North America, parents are thought to wield greater power during childhood; however, with the onset of adolescence, the parent-child power dynamic becomes less vertical and more balanced [28]. Additionally, in North America, it is thought that there is a bias toward the socialization of independence by encouraging autonomy, assertiveness and self-reliance from the early years of childhood [28]. Furthermore, parents usually value warmth, non-punitive methods and behavioral control in their childrearing practices, and greater significance is attributed to extra-familial relationships for social provisions and support [7].

In contrast, Southern societies, including LatinX cultures, have been typically characterized by more collectivist norms and values [29]. In these cultural contexts, the power structure between parents and children is thought to remain asymmetrical throughout childhood and early adolescence [28]. Thus, there is purported to be a greater emphasis on conformity, compliance, respect for authority and interdependence in social relationships [28].

Given that social relationships are defined and regulated by cultural norms and values, there is clearly a need to consider how parent-child relationships and friendships are manifested in various cultures and how the underlying constructs or provisions of these relationships are perceived and evaluated by individuals within different cultures [7]. The majority of empirical studies that have explored cultural differences in relationship qualities, using similar sets of items, have been conducted in Asian [30,31,32,33] and African societies [34,35]. Less is known about the perceived relationship qualities among adolescents from LatinX cultures [36,37].

Like Central and Southern American LatinX societies, Portuguese society continues to be regulated by collectivist and family-oriented values that establish strong family responsibilities and expectations [38], a high interdependency between the family members [39,40] and an emphasis on the values of respect and authority [41]. However, traditional norms and values coexist with an increasing diffusion of individualistic values in Portugal [40]. Although family relationships in contemporary Portugal continue to be strong, it is also the case that, among the youngest generations, the role of extra-familial relationships, such as friendship, have become of increasing significance [39,40]. These more contemporary developments may influence how the underlying constructs or provisions of close relationships are perceived and evaluated by Portuguese adolescents.

### 1.3. The Present Study

To the best of our knowledge, researchers have yet to examine Portuguese young adolescents’ perceptions of their relationships with their mothers, fathers and best friends when compared with North American youth, using the same set of items to comprehensively understand relationship constructs. Furthermore, the replicability of previous associations between perceived relationship qualities and satisfaction [7] in Southern European societies (especially, Portugal) remains unexplored.

Due to the existing gaps in the current state-of-art knowledge, the primary aims of the present study were to: (1) compare the perceived provisions and satisfaction of USA and Portuguese adolescents in their relationships with parents and best friends and (2) examine the influence of perceived relationship qualities on the satisfaction of young adolescents in their relationships with mothers, fathers and best friends.

Based on the extant literature, we expected that USA young adolescents would perceive higher levels of support and satisfaction in their relationships with their best friends than in their relationships with parents. In contrast, we expected that Portuguese young adolescents would perceive higher levels of support and satisfaction in their relationships with their parents than in their relationships with their friends.

With respect to negativity, we expected that USA young adolescents would perceive higher levels of negativity in their relationships with parents and lower levels of negativity in their relationships with same-sex best friends when compared with Portuguese young adolescents. Relatedly, we expected that USA young adolescents would perceive lower levels of power in their relationships with parents and friends when compared with Portuguese young adolescents.

In both countries, we expected that higher levels of support and lower levels of negativity would be associated with lower levels of satisfaction with close relationships. In the USA, we expected that adolescents would be dissatisfied with the relationship if they perceived their parents as attempting to maintain a vertical power relationship. In Portugal, we expected that the maintenance of a vertical parent-child power relationship would not be associated with dissatisfaction.

## 2. Materials and Methods

### 2.1. Participants

The sample consisted of 347 USA adolescents (170 boys, 177 girls) and 360 Portuguese adolescents (176 boys, 184 girls). USA adolescents (*M* = 13.64, *SD* = 0.56) and Portuguese adolescents (*M* = 13.60, *SD* = 0.78) were of comparable ages, *t* = 0.794, *p* = 0.427, *d* = 0.06. The USA sample was ethnically diverse, with participants self-identifying as European-American (56%), Asian-American (19%), Latino/Hispanic (10%), African-American (8%) or bi-/multi-racial (7%). Due to legal restrictions in Portugal, data on ethnicity were not collected in Portugal. Inclusion criteria were as follows: (1) attending 7th to 9th grade; (2) residing in each nation’s capital region; and (3) being able to read and understand each nation’s language. Young adolescents with learning disabilities were excluded.

### 2.2. Procedures 

Portugal. In the Portuguese sample, the study’s aims and procedures were presented to three boards of public junior high schools from the Metropolitan Lisbon (7th to 9th grade) from middle class neighborhoods in order to obtain their authorization to collect the data. After the school boards’ approval, parents/legal guardians of all adolescents were asked to sign a written informed consent from to authorize adolescents’ participation in the study. Before data collection, participants were informed about the aims and procedures of the study, the voluntary nature of their participation and the confidentiality of their responses. In addition to the written consent of parents/legal guardians, researchers requested adolescents’ assent. The refusal of the minor to provide assent to participate in the present study was respected. After performing these ethical procedures, two trained researchers introduced the study and administered the measures, in regular school hours, in class, during a single 45-min session.

USA. Participants were recruited from a larger sample of adolescents who had previously participated in a wider longitudinal study. Participants attended public schools in the Metropolitan Washington DC area. All participants were first contacted by telephone, and, if both parents and adolescents expressed interest, parental consent and adolescent assent forms were mailed to the home with pre-addressed and stamped return envelopes. Adolescents were given the option of completing questionnaires on paper or online. Depending on participant preference, packets of questionnaires were mailed home (approximately 80% of the sample) or a link to a secure website was sent via email (20% of the sample).

### 2.3. Measures

*Network of Relationships Inventory Social Provision Version* (NRI-SPV, [12]): The NRI-SPV consists of 33 items that assess adolescents’ perceptions of relationship qualities and satisfaction concerning six types of close relationships: (1) mother or stepmother, (2) father or stepfather, (3) best friend, (4) teacher, (5) other relatives and (6) siblings (from the first to the fourth one). In this questionnaire, participants were asked to rate how much or how frequently each of the presented statements describe their relationships with each of the previously identified persons, using a Likert scale from 1 (*None/Not at all*) to 5 (*Very Much/Almost Always*). For the aims of the present study, we only considered adolescents’ ratings concerning their relationships with their mothers, fathers and best friends. Following the recommendations of prior research [12,15,17], the mean scores of the two higher-order factors (Social Support and Negativity) of the NRI-SPV were calculated. Social Support encompasses seven subscales assessing supportive relationship provisions: (1) Reliable Alliance (three items; e.g., *“How sure are you that this relationship will last no matter what?*”), (2) Admiration (three items; e.g., *“How much does this person treat you like you’re admired and respected?”*), (3) Affection (three items; e.g., *“How much does this person like or love you?”*), (4) Companionship (three items; e.g., *“How often do you spend fun time with this person?”*), (5) Instrumental Aid (three items; e.g., *“How much does this person teach you how to do things that you don’t know?”*), (6) Intimate Disclosure (three items; e.g., *“How often do you tell this person things that you don’t want others to know?”*) and (7) Nurturance (three items; e.g., *“How much do you help this person with things she/he can’t do by her/himself?*”). Conversely, Negativity encompasses the subscales of Conflict (three items, e.g., *“How often do you and this person disagree and quarrel with each other?”*) and Antagonism (two items, e.g., *“How much do you and this person hassle or nag one another?”*). In addition, the mean scores of Relative Power (three items, e.g., *“Who tells the other person what to do more often, you or this person?”*) and Satisfaction (three items, e.g., *“How satisfied are you with your relationship with this person?”*) were considered for the aims of the present study. Higher mean scores of Support, Negativity, Relative Power and Satisfaction indicate greater perceived supportive relationship provisions, relationship-straining features, power imbalance and satisfaction in the relationships with mothers, fathers and best friends. In our sample, Cronbach’s alphas ranged from 0.64 (Relative Power—Best Friend) to 0.93 (Satisfaction—Father).

### 2.4. Data Analysis

The data analysis was computed using IBM Statistics 27. To compare the perceived Social Support, Negativity, Relative Power and Satisfaction of USA and Portuguese adolescents in their relationships with their mothers, fathers and same-sex best friends, a mixed analysis of covariance (ANCOVA) was performed, using country as a between-subject factor, the type of relationship as a within-subject factor and sex as a covariate. When a significant effect was found, pairwise comparisons with Bonferroni corrections were conducted.

Following the procedures recommended by [42], moderated linear regression analyses were performed to examine the predictive role of the perceived qualities in the relationships with mothers, fathers and same-sex best friends in satisfaction with each of these relationships, depending on country. After inserting sex as a covariate in the first step, the study variables (which were centered to control multicollinearity) and the moderator (country; dummy-coded 0 = Portugal and 1 = USA) were introduced. In the last step, interaction terms were introduced. When a significant interaction was found, post-hoc simple slopes analyses were performed using Modgraph. The significance level was set at *p* < 0.05.

## 3. Results

### 3.1. Perceived Relationship Provisions of USA and Portuguese Adolescents with Parents and Best Same-Sex Friends

Table 1 presents the comparisons of perceptions of relationship qualities and satisfaction with mothers, fathers and same-sex best friends, depending on country and sex.

#### 3.1.1. Perceived Social Support

Table 1 indicates that a significant Type of Relationship × Country interaction effect was found. Portuguese and USA adolescents perceived higher support in their relationships with their mothers and same-sex best friends than in their relationships with their fathers. However, the magnitude of the differences in perceived social support was higher for Portuguese adolescents. A significant main effect of country was also found. USA adolescents perceived higher levels of Support than Portuguese adolescents. Furthermore, a significant main effect of the type of relationship was found. Adolescents perceived lower Support in their relationships with their fathers than in their relationships with their mothers and same-sex best friends.

A significant Type of Relationship × Sex was found. Girls perceived higher Support in their relationships with their mothers than with their fathers; however, girls also perceived higher Support in their relationships with their same-sex best friends than in their relationships with both parents. In contrast, boys perceived higher Support in their relationships with their same-sex best friends than in their relationships with their fathers; however, boys perceived higher Support in their relationships with their mothers than in their relationships with their fathers and their same-sex best friends. No significant main effect of sex was found.

#### 3.1.2. Perceived Negativity

Table 1 shows that a significant Type of Relationship × Country was found. Portuguese adolescents perceived higher levels of Negativity in their relationships with their mothers than with their fathers and same-sex best friends. In addition, Portuguese and USA adolescents perceived higher levels of Negativity in their relationships with their fathers than with their best friends. However, the magnitude of the difference between the perceived Negativity in the relationships with parents and same-sex best friends was higher in the USA than in Portugal.

A significant main effect of the type of relationship was found. Adolescents perceived greater Negativity in their relationships with both their mothers and fathers than with their best friends. In addition, adolescents perceived greater Negativity in their relationships with their mothers than with their fathers. A significant main effect of country was also found. USA adolescents perceived higher levels of Negativity than Portuguese adolescents.

A significant Type of Relationship × Sex effect was found. Both girls and boys perceived higher Negativity in their relationships with their mothers than with their fathers and same-sex best friends. In addition, girls and boys perceived higher Negativity in their relationships with their fathers than their best friends. However, the magnitude of the difference in the perceived Negativity in the relationships with mothers and fathers was lower for boys than for girls. No significant main effect of sex was found.

#### 3.1.3. Perceived Power

Table 1 shows that a significant Type of Relationship × Country was found. Portuguese and USA adolescents perceived higher levels of Power in relationships with their parents than with their same-sex best friends. In addition, Portuguese and USA adolescents perceived higher levels of Power in the relationships with their mother than with their fathers. The magnitude of the difference in perceived Power in the relationships with mothers and fathers was higher for Portuguese adolescents than for USA adolescents. The magnitude of the difference in perceived Power in the relationships with parents and same-sex best friends was lower for Portuguese than for USA adolescents.

A significant main effect of country was found. Portuguese adolescents perceived higher levels of Power in their relationships than USA adolescents. A significant main effect of type of relationship was also found. Adolescents perceived higher levels of Power in their relationships with their parents than with their same-sex best friends. In addition, adolescents perceived higher levels of Power in their relationships with their mothers than with their fathers.

A significant Type of Relationship × Sex was found. Girls and boys perceived higher levels of Power in their relationships with their parents than with their same-sex best friends. In addition, girls and boys perceived higher levels of Power in their relationships with their mothers than with their fathers, but the magnitude of these differences was higher for girls than for boys. No significant main effect of sex was found.

#### 3.1.4. Perceived Satisfaction

Table 1 reveals that there were no significant main effects of country. A significant main effect of type of relationship was found. Adolescents perceived higher levels of Satisfaction in their relationships with their mothers and same-sex best friends than in their relationships with their fathers. However, no significant interaction Type of Relationship × Country was found.

A significant main effect of sex was found. Boys perceived higher levels of Satisfaction in their relationships than girls. Furthermore, a significant Type of Relationship × Sex was found. Girls and boys perceived higher levels of Satisfaction in their relationships with their mothers than in the relationships with their fathers. Girls perceived higher levels of Satisfaction in their relationships with their same-sex best friends than in their relationships with their parents. However, boys only perceived higher levels of Satisfaction in their relationships with their same-sex best friends than in their relationships with their fathers. In contrast, boys perceived higher levels of Satisfaction in their relationships with their mothers than in their relationships with their same-sex best friends.

### 3.2. Perceived Relationship Qualities and Satisfaction of Young Adolescents’ Relationships with Mothers, Fathers and Best Friends

#### 3.2.1. Relationship with Mothers

**Figure 1:**
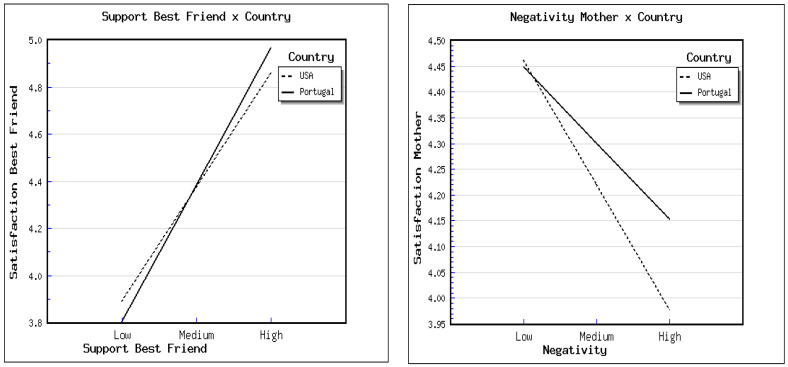
The influence of perceived negativity in the relationship with the mother and of perceived support from the same-sex best friend in perceived relationship satisfaction, depending on country.

Table 2 shows that higher levels of Support and lower levels of Negativity were associated with higher levels of satisfaction with the mother-child relationship. Furthermore, boys and Portuguese youth also reported higher levels of Satisfaction with their mother-child relationships. The introduction of Perceived Negativity × Country significantly improved the explained variance of the model. Figure 1 shows that the Satisfaction with the mother-child relationship significantly decreased as Negativity increased in the USA (*t* = −6.93, *p* < 0.001) but not in the Portuguese (*t* = −6.01, *p* = 0.380) sample.

#### 3.2.2. Relationship with Fathers

Table 2 shows that higher levels of Support and lower levels of Negativity were associated with higher levels of Satisfaction with the father-child relationship. Furthermore, boys and Portuguese youth also reported higher levels of Satisfaction with the father-child relationship. The introduction of interaction terms did not significantly improve the explained variance of the model.

#### 3.2.3. Relationship with Same-Sex Best Friends

Table 2 shows that boys reported higher levels of perceived Satisfaction with the relationship with same-sex best friends. Furthermore, higher levels of Support and lower levels of Negativity were associated with higher levels of Satisfaction with the relationship with same-sex best friends. The introduction of Perceived Support × Country significantly improved the explained variance of the model. Figure 1 shows that the increase in Satisfaction as Support increased was higher in Portugal (*t* = 27.16, *p* < 0.001) than in the USA (*t* = 16.77, *p* < 0.001).

## 4. Discussion

The aims of this study were to compare the perceived relationship qualities of USA and Portuguese adolescents with their parents and best friends and to examine the influence of these qualities on relationship satisfaction.

Perceived relationship qualities in USA and Portugal. Contrary to our hypothesis, adolescents from *both* countries described mothers and best friends as equally supportive and as providers of higher levels of support than fathers. These findings are in line with prior research conducted in the USA [17,18] and Southern Europe [43] showing that mothers typically share the position of the most frequent providers of support with best friends during early adolescence. Furthermore, these findings are consistent with changes that occur during the transition to adolescence toward a greater reliance on friends for emotional support [17].

The similarities in the perceived levels of support and satisfaction provided by mothers and best friends in the Portuguese sample may also reflect the heightened significance of friends among the youngest generations as providers of support [40]. In spite of these generational changes, [44] suggested that Southern European adolescents are typically confronted with a greater intrapersonal conflict between being autonomous and remaining closely connected to parents than adolescents from more individualistic cultures. Furthermore, parental autonomy-support has been found to satisfy some of children’s basic psychological needs and ameliorate social adjustment [45]. Although the differences were low in magnitude, the greater intrapersonal conflict between being autonomous and remaining closely connected to parents may explain why Portuguese adolescents globally perceived their close relationships to be less supportive than USA adolescents.

As expected, USA adolescents reported higher levels of satisfaction in their relationships with friends than in their relationships with parents. The ranking of scores vis-à-vis satisfaction in our study differ from the results of US studies conducted in the decade of 1990 [12,18]. However, the findings reported herein may reflect generational, historical changes, suggesting that North American cultural norms now place a greater significance on extra-familiar relationships for social provisions and support [7,27].

On the other hand, our findings are in line with prior research [12,17,19] showing that adolescents typically perceive their relationships with parents to be more negative (i.e., conflictual and punitive) and imbalanced in power than their relationships with friends. As hypothesized, the magnitude of the difference in perceived negativity depending on the type of relationship (parents vs. best friends) was greater for USA adolescents than for Portuguese adolescents. Indeed, North American cultures typically encourage autonomy and self-reliance [28] such that the expected initial peaks of tension in parent-child relationships [12,19] occur earlier and are more intense among USA adolescents. Although the differences were low in magnitude, the heightened perceptions of power imbalances in close relationships among Portuguese young adolescents are consistent with the notion that traditional norms valuing respect and authority [41] continue to coexist with individualistic values in the Portuguese society [40].

Lastly, our findings seem to support the notion that mothers are typically a greater supportive presence and are committed to the supervision of their adolescents’ daily lives [43], so youth in both cultures perceived their relationships with their mothers as being more supportive and satisfying but also more conflictual and vertical in power balance than their relationships with their fathers. Nevertheless, these differences in perceived relationship qualities between mothers and fathers were more salient in Portugal. [14] showed that nearly 30% of Portuguese youth were classified in low father support profiles. Furthermore, prior empirical studies have shown that Portuguese fathers continue to be less involved in the daily responsibilities of parental care than mothers [46,47], possibly explaining the greater differences in perceived relationship qualities between Portuguese mothers and fathers.

Sex differences in perceived relationship qualities. Our findings revealed that girls perceived their same-sex best friendships as being more supportive, less negative and more satisfactory than their relationships with parents. This was not the case for boys. These findings may be explained by the earlier pubertal timing among girls, which can accelerate the development of autonomy from parents [19]. The more favorable perceptions of girls toward same-sex best-friend relationships are also consistent with prior research [2,12,15,20,21] and may reflect the preference of girls for more intense dyadic relationships when compared with boys, who typically prefer extensive peer group relationships [12,48].

With respect to power, our findings revealed that girls and boys perceived that the power dynamic was more imbalanced in their relationships with their parents (especially with their mothers) than in their relationships with their friends. This magnitude of difference was higher for girls, possibly supporting the notion that parents may continue to foster traditional sex-role stereotypes in power balance [17].

Predicting relationship satisfaction. In line with prior studies conducted in USA [7] and Asian [15] samples, our findings showed that higher levels of perceived support and lower levels of negativity were associated with higher levels of perceived satisfaction within close relationships in both countries. The findings, however, did demonstrate some low-magnitude country differences in the concomitants of relationship satisfaction.

With respect to mother-young adolescent relationships, our findings indicated that the negative association between perceived negativity and satisfaction was stronger in the USA than in Portugal. In spite of its low magnitude, the stronger influence of perceived negativity on satisfaction with the mother-young adolescent relationship in the USA may be explained by the cultural bias toward the early socialization of independence [25,28]. Given these cultural norms and values, maternal opposition to, or interfering with, the expression of adolescent behaviors may be viewed more negatively by USA adolescents and thereby lead to less satisfaction in the mother-adolescent relationship. Conversely, more controlling child-rearing practices and parental guidance and a lower focus on the promotion of independence are considered normative parental behaviors in Portugal [49,50]. Thus, these family-oriented values may attenuate the negative association between perceived negativity and satisfaction with mother-adolescent relationships.

Turning to friendship, the positive association between support and satisfaction was stronger in Portugal than in the USA. These findings may reflect the greater emphasis on interdependence in social relationships in LatinX-oriented cultures [27,33]. This may be especially important in Portugal, where support is viewed as one of the major dimensions of a good friendship [51]. Thus, cultural norms and values may explain why perceived support has a stronger influence on the friendship satisfaction of Portuguese than American young adolescents.

Strengths and limitations. To the best of our knowledge, this was the first study to compare the perceived relationship provisions of USA and Portuguese adolescents and the influence of these provisions on relationship satisfaction with mothers, fathers and same-sex best friends. From a methodological standpoint, the use of the same set of items to examine, comprehensively, cross-cultural similarities and differences in relationship provisions and satisfaction [12] represents an advance in the current state-of-the-art knowledge pertaining to young adolescents’ significant relationships.

However, some limitations must be acknowledged. In both samples, participants were recruited using a convenience sampling method in only three schools in Metropolitan Lisbon and three schools in the Greater Washington, DC area, thereby somewhat limiting the generalization of the findings. Furthermore, our cross-sectional data set did not make it possible to establish causal relationships between the various indices of relationship provision and relationship quality (satisfaction). Future empirical studies would do well to address these methodological limitations, using longitudinal designs to assess the relationship provisions that predict relationship satisfaction. From a methodological standpoint, the cross-cultural validation of the *Network of Relationships Inventory* (NRI), using multi-group confirmatory factor analyses, would also represent an advance in the current state-of-art knowledge. Further cross-cultural studies on family-peer relationships are needed [52] to explore the perceived qualities and satisfaction in close relationships among adolescents from a larger number of countries. Given that recent studies highlight the complex influences of friends and parents on the development of psychosocial difficulties during adolescence [53], the interaction between perceived qualities in the relationships with parents and best friends on adolescents’ perceived satisfaction in close relationships needs to be explored. Lastly, the mechanisms that may explain cultural and sex differences (e.g., parental autonomy-support [45]) in adolescents’ perceived qualities and satisfaction in close relationships) also require closer inspection.

## 5. Conclusions

The findings of the present study support that young adolescents’ perceived qualities and satisfaction in close relationships may differ depending on cultural norms. Significantly, our findings also have some implications for intervention. Due to the stronger relation between perceived negativity and satisfaction with the mother-adolescent relationship, a psychoeducational approach to the use of appropriate parenting strategies could be useful to enhancing the use of non-punitive and appropriate behavioral control. On the other hand, the stronger association between perceived relationship support and satisfaction with same-sex best friends suggests that it may be useful to promote training on socioemotional competencies that can enhance the formation and maintenance of supportive friendships.

## Figures and Tables

**Table 1:** Comparisons of the perceptions of relationship qualities and satisfaction with mothers, fathers and same-sex best friends, depending on country and sex.

	USA	Portugal	Girls	Boys	Relationship Type × Country				Country		Relationship Type		Sex		Relationship Type × Sex	
	*M* (*SD*)	*M* (*SD*)	*M* (*SD*)	*M* (*SD*)	*F*	η^2^_p_	USA	Portugal	*F*	η^2^_p_	*F*	η^2^_p_	*F*	η^2^_p_	*F*	η^2^_p_
Social Support					5.26 **	0.008	Mo > Fa *** Fr > Fa ***	Mo > Fa *** Fr > Fa ***	13.47 ***	0.019	79.77 ***	0.103	1.16	0.002	20.94 ***	0.051
Mother	3.97 (0.70)	3.92 (0.77)	3.94 (0.86)	3.95 (0.62)												
Father	3.70 (0.76)	3.43 (1.11)	3.49 (0.98)	3.62 (0.94)												
Best friend	3.99 (0.57)	3.86 (0.73)	4.07 (0.61)	3.77 (0.68)												
Negativity					107.98 ***	0.135	Mo > Fa ** Mo > Fr *** Fa > Fr ***	Mo > Fa *** Mo > Fr *** Fa > Fr ***	0.19	0.000	258.72 ***	0.272	0.13	0.000	9.13 ***	0.013
Mother	2.96 (0.75)	2.78 (0.82)	2.93 (0.80)	2.81 (0.78)												
Father	2.82 (0.86)	2.44 (0.87)	2.57 (0.89)	2.69 (0.88)												
Best friend	1.67 (0.52)	2.16 (0.66)	1.94 (0.69)	1.90 (0.61)												
Power					57.84 ***	0.077	Mo > Fa ** Mo > Fr *** Fa > Fr ***	Mo > Fa *** Mo > Fr *** Fa > Fr ***	26.62 ***	0.037	307.65 ***	0.307	0.01	0.000	5.25 **	0.008
Mother	3.82 (0.90)	4.01 (0.74)	3.95 (0.83)	3.88 (0.83)												
Father	3.67 (0.87)	3.54 (1.26)	3.53 (1.12)	3.67 (1.06)												
Best Friend	2.21 (0.67)	2.92 (0.87)	2.59 (0.91)	2.55 (0.81)												
Satisfaction					2.84	0.004			1.74	0.187	55.43 ***	0.074	5.05 *	0.007	10.72 ***	0.015
Mother	4.28 (0.89)	4.36 (0.95)	4.21 (1.02)	4.43 (0.79)												
Father	3.96 (1.10)	3.87 (1.35)	3.80 (1.28)	4.02 (1.19)												
Best Friend	4.46 (0.62)	4.30 (0.73)	4.44 (0.65)	4.32 (0.72)												

Mixed ANOVAs followed by post-hoc comparisons with Bonferonni corrections. Note. Mo means Mother. Fa means Father. Fr means Best Friend. *** *p* < 0.001, ** *p* < 0.01, * *p* < 0.05.

**Table 2:** Final regression models examining the predictive role of the perceptions of relationships with the mothers, fathers and same-sex best friends in satisfaction, depending on country.

	Perceived Satisfaction in the Relationship with the Mother				Perceived Satisfaction in the Relationship with the Father				Perceived Satisfaction in the Relationship with Same-Sex Best Friend			
	**β**	***t***	***F***	***R* ^2^**	**Β**	***t***	***F***	***R* ^2^**	**β**	***t***	***F***	***R* ^2^**
			212.28 ***	0.71			558.48 ***	0.79			167.27 ***	0.66
Sex ^a^	0.10	4.96 ***			0.04	2.44 *			0.08	3.52 ***		
Perceived Support	0.75	21.75 ***			0.89	24.17 ***			0.31	33.28 ***		
Perceived Negativity	−0.17	−5.15 ***			−0.11	−3.85 ***			−0.16	−4.96 ***		
Perceived Power	0.02	0.61			0.02	0.51			0.02	0.46		
Country ^b^	−0.05	−2.22 **			−0.04	−2.33 *			−0.009	−0.34		
Country × Perceived Support	0.03	0.96							−0.09	−2.77 **		
Country × Perceived Negativity	−0.07	−2.14 *							0.02	0.58		
Country × Perceived Power	−0.04	−0.11							−0.06	−1.60		

Note. Interaction terms are not presented for the model of fathers, because these terms did not improve the percentage of explained variance. ^a^ Dummy-coded as: 1—male, 0—female. ^b^ Dummy-coded as: 1—USA, 0—Portugal. *** *p* < 0.001, ** *p* < 0.01, * *p* < 0.05.

## Data Availability

The data that support the findings of this study are available on request from the corresponding author. The data are not publicly available due to privacy and ethical restrictions.
